# Prediction of plantar soft tissue stiffness based on gender, age, bodyweight, height and body mass index

**DOI:** 10.1186/1757-1146-7-S1-A128

**Published:** 2014-04-08

**Authors:** Jee Chin Teoh, Taeyong Lee

**Affiliations:** 1 Department of Biomedical Engineering, National University of Singapore, Singapore

## Introduction

Stiffened plantar soft tissues break down easily (Cheung et al., 2005) and these microscopic tears will heap together and develop into a large ulcer. In the USA, 85% of all non-traumatic amputations in diabetes patients arise from non-healing ulcers (Larsson, 1994). In fact, foot ulceration is one of the major causes of hospitalization among the DM patients. 15% of the DM population are threatened by high ulceration risk during their life time (Aziz nather’s book). These findings elucidate the need of early identification of degenerating plantar soft tissue to prevent problematic tissue rupture, especially to diabetic and elderly patients. Non-invasive in vivo assessment that enables direct measurement of tissue’s mechanical response is therefore required. In order to differentiate between normal and pathological tissue, a stiffness reference is needed.

The objective of this study is to conduct a multivariate analysis on the data of plantar tissue stiffness to a better understanding on the influences and the use of these parameters to predict the healthy tissue stiffness of these individuals.

## Methods

100 healthy subjects were recruited from National Seoul University (SNU) hospital for the experiment.

During stiffness measurement [1], indentor tip probes the plantar soft tissue to obtain localized force response underneath the 2nd metatarsal head pad at 3 different dorsiflexion angles of 0°, 20°, 40° and the hallux and heel at 0°. Maximum tissue deformation is fixed at 5.6mm (close to literature data) [2].

Tissue behavior was characterized via K, stiffness constant.

Multiple linear regression of soft tissue stiffness was performed on several plantar locations. The independent variables are gender (-1 for females and +1 for males), bodyweight, height, BMI and age. Multiple analysis was chosen to study the combined effects of the independent variables on tissue stiffness.

## Results

However, moderately strong relationship was found on the combined effects of these independent variables as shown in Table 1.

**Table 1:** *Linear regression equation: partial regression coefficients*, *p values*, *root mean square errors* (RMSE) and number of participants.Y= b0 + b1 x gender + b2 x weight + b3 x height + b4 x bmi + b5 X age; b0 (n/mm); b1 (n/mm)/gender, female= -1, male = +1; b2 (n/mm)/weight; b3 (n/mm)/height; b4 (n/mm)/bmi; b5 (n/mm)/age

Plantar location		b0	Gender (b1)	p	Weight (b2)	p	Height (b3)	p	BMI (b4)	p	Age (b5)	p	RMSE	n
***Left***														
Hallux		-1.8669817	-0.10225389		-0.0042387		0.01183471		-0.0133015		0.02399122		0.48578	99
Heel		12.3844196	-0.12954317		0.08095755	*	-0.0619824	*	-0.1717064	*	0.00629858		1.09547	100
2^nd^MTH	0°	6.46521444	-0.2177458		0.06521518	**	-0.0443825	*	-0.152051	**	0.02342962		0.802866	96
	20°	8.58233676	-0.37727232		0.11675474	**	-0.0669571	**	-0.2698104	**	0.05478136		0.989617	100
	40°	12.5772582	-0.15231289		0.12385643	**	-0.0855753	*	-0.3131397	**	0.06469774		1.479414	98
***Right***														
Hallux		0.66899629	0.087890718		-0.0165869		0.00670808		0.03648811		-0.0093199		0.409141	99
Heel		17.6691906	-0.54480677	*	0.14765975	**	-0.0882361	**	-0.3480107	**	-0.0132202		1.147579	100
2^nd^MTH	0°	4.48332802	-0.17447007		0.04778213	*	-0.0271027		-0.1134243	*	0.01582428		0.821476	96
	20°	14.2540307	-0.34137115		0.12512617	**	-0.0797136	**	-0.2852428	**	-0.0020648		1.126962	100
	40°	16.389124	-0.20310698		0.13467015	**	-0.0842629	*	-0.2962142	**	-0.0117724		1.614592	98

This suggest that the decision to ignore the influence of gender, age, bodyweight, height and body mass index on plantar soft tissue stiffness should be carefully considered. The combined effect of these independent parameters may subtly influence the accuracy of the study analysis.
